# Modeling the obesity epidemic: social contagion and its implications for control

**DOI:** 10.1186/1742-4682-10-17

**Published:** 2013-03-09

**Authors:** Keisuke Ejima, Kazuyuki Aihara, Hiroshi Nishiura

**Affiliations:** 1 School of Public Health, The University of Hong Kong, Level 6, Core F, Cyberport 3, Pokfulam, Hong Kong; 2 Department of Mathematical Informatics, Graduate School of Information Science and Technology, The University of Tokyo, 7-3-1 Hongo, Bunkyo-ku, Tokyo, 113-8656, Japan; 3 Institute of Industrial Science, The University of Tokyo, 4-6-1 Komaba, Meguro-ku, Tokyo, 153-8505, Japan; 4 PRESTO, Japan Science and Technology Agency, Saitama, Japan

## Abstract

**Background:**

As an obesity epidemic has grown worldwide, a variety of intervention programs have been considered, but a scientific approach to comparatively assessing the control programs has still to be considered. The present study aims to describe an obesity epidemic by employing a simple mathematical model that accounts for both social contagion and non-contagious hazards of obesity, thereby comparing the effectiveness of different types of interventions.

**Methods:**

An epidemiological model is devised to describe the time- and age-dependent risk of obesity, the hazard of which is dealt with as both dependent on and independent of obesity prevalence, and parameterizing the model using empirically observed data. The equilibrium prevalence is investigated as our epidemiological outcome, assessing its sensitivity to different parameters that regulate the impact of intervention programs and qualitatively comparing the effectiveness. We compare the effectiveness of different types of interventions, including those directed to never-obese individuals (i.e. primary prevention) and toward obese and ex-obese individuals (i.e. secondary prevention).

**Results:**

The optimal choice of intervention programs considerably varies with the transmission coefficient of obesity, and a limited transmissibility led us to favour preventing weight gain among never-obese individuals. An abrupt decline in the prevalence is expected when the hazards of obesity through contagious and non-contagious routes fall into a particular parameter space, with a high sensitivity to the transmission potential of obesity from person to person. When a combination of two control strategies can be selected, primary and secondary preventions yielded similar population impacts and the superiority of the effectiveness depends on the strength of the interventions at an individual level.

**Conclusions:**

The optimality of intervention programs depends on the contagiousness of obesity. Filling associated data gaps of obesity transmission would help systematically understand the epidemiological dynamics and consider required control programs.

## Background

Obesity has become more and more widespread, increasingly recognized as one of the biggest global health problems. According to the estimate of the World Health Organization (WHO), the prevalence of obese individuals across the world was estimated at 9.8% in 2005 [1], and with a subsequent increase, an urgent preventive action has been deemed essential. The public health need for obesity control is evident, because obesity serves as one of the most important risk factors of various chronic diseases [2], including acute coronary heart disease and other circulatory diseases, diabetes and several types of cancer (e.g. colon cancer). Following the WHO’s declaration of the global epidemic of obesity in 1997 [2], the World Health Assembly endorsed the Global Strategy on Diet, Physical Activity and Health (DPAS) in 2004 aiming to improve the situation by intervening diet and physical activity [3]. Accordingly, the member states of the WHO and other international partners have faced a need to construct and carry out obesity control programs. As part of the control effort, various epidemiological studies have been conducted to assess the effectiveness of each control program (i.e. through individual nutritional or physical exercise programs). However, there have been little attempt to qualitatively and quantitatively compare the effectiveness of different types of control programs and optimize obesity control program as a whole. In addition, very little epidemiological effort has been made to understand the entire epidemiological dynamics of obesity and its control using mathematical and theoretical approaches.

While actual interventions of dietary behaviours (e.g. avoiding excessive calorie intake) and those against insufficient physical activities are implemented, Christakis and Fowler [4] scientifically demonstrated that obesity can spread from person to person via a social contact network. The epoch-making finding of the spread of non-infectious disease through a social contact network was not only limited to obesity but also other health-related issues such as smoking [5]. Statistical review of social network analysis took place elsewhere [6], because the estimation problem of social network effects, including the use of dynamic models and statistical control of confounders, has been discussed [7,8]. The underlying biological and social mechanisms of obesity epidemics have fascinated a broad range of scientific audience.

Provided that non-negligible fraction of obesity is caused by person-to-person transmission, the effectiveness of essential control programs against obesity epidemic would be characterized by nonlinear dynamics with a correlated risk structure. That is, estimating the risk of obesity involves the issue of dependence in which the risk of obesity in a single individual is determined not only by that particular individual but also by other individuals in the same population unit (i.e. the so-called “dependent happening”). In a positive sense, the dependence implies that one could expect herd effect (or herd immunity) by implementing public health interventions, which has been commonly seen in the epidemiology of infectious diseases [9]. However, it also implies that the contagious effect could lead to social problems including potential need to intervene friendship network and social discrimination.

The present study aims to describe an obesity epidemic by employing a simple mathematical model that accounts for both social contagion and non-contagious hazards of obesity, thereby comparing the effectiveness of different types of interventions. Using a simplistic model with randomly mixing assumption, we intend to explore the most effective intervention in a qualitative manner and identify epidemiological data gaps that have prevented us from explicitly evaluating and comparing the effectiveness of various obesity control programs.

## Methods

### A model for the social contagion of obesity

Considering that obesity is caused by both contagious and non-contagious routes, we describe the epidemiological process of becoming and recovering from obesity as a function of time. Despite the fact that the spread of obesity is believed to occur on a complex social network [4,6], here we exploit a model that describes the epidemiological process of obesity in a randomly mixing population, because the present study intends to clarify the implications of person-to-person transmission of obesity for public health control in a rudimentary fashion and identify fundamental data gaps that have to be urgently addressed in empirical observations. To describe the time-dependence of the risk of obesity, we use the ordinary differential equations (ODE) that capture the population dynamics of obesity. Referring to the simplest version of the most classical epidemiological model for directly-transmitted infectious diseases [10,11], we describe the time-evolution of susceptible (never-obese), infectious (obese) and recovered (ex-obese) individuals as a function of time *t*, namely, *S*(*t*), *I*(*t*) and *R*(*t*) as follows:

(1) $$\begin{array}{c} \frac{dS}{dt}=\mathrm{\mu N}-\left[\mathrm{\beta I}\left(t\right)+\varepsilon \right]S\left(t\right)-\mathrm{\mu S}\left(t\right),\\ \frac{dI}{dt}=\left[\mathrm{\beta I}\left(t\right)+\varepsilon \right]S\left(t\right)+\sigma \left[\mathrm{\beta I}\left(t\right)+\varepsilon \right]R\left(t\right)-\left(\mu +\gamma \right)I\left(t\right),\\ \frac{dR}{dt}=\mathrm{\gamma I}\left(t\right)-\sigma \left[\mathrm{\beta I}\left(t\right)+\varepsilon \right]R\left(t\right)-\mathrm{\mu R}\left(t\right), \end{array}$$

where *N* represents the total population size, assumed to be a constant over time for the sake of our exposition of epidemiological data gaps, that is, *N* = *S*(*t*) + *I*(*t*) + *R*(*t*) for any *t*, *μ* is the birth and death rate of human host, *β* is the transmission coefficient, *ε* is the hazard of obesity due to non-contagious reasons, *γ* is the natural recovery rate, and *σ* is the relative risk of weight regain among ex-obese individuals which typically takes a value greater than 1 due to high risk of coming back to the obese state [12]. It should be noted that the system (1) assumes that ex-obese is not contagious. All three equations describe the background birth and death of the host using the rate, *μ*. Otherwise all terms are associated with acquirement of or recovery from obesity. Among never-obese individuals, *λ*(*t*) = *βI*(*t*) + *ε* is the hazard rate of obesity on a whole (or, is frequently referred to as the “force of infection” in infectious disease epidemiology) at which they experience obesity for the first time. Among ex-obese individuals, the hazard is *σ* times greater than that among never-obese individuals. The natural recovery of obesity occurs at the rate, *γ*.

It should be noted that the force of infection, *λ*(*t*) is modelled in an additive manner, i.e., expressed as a sum of two hazards, one through the contagious route *λ*_1_ = *βI*(*t*) and the other via the non-contagious route *λ*_2_ = *ε*, the latter of which is determined by many factors including genetics and lifestyle including dietary habit. For simplicity, we consider a situation in which *λ*_2_ is constant. By employing the additive model for the force of infection, it is assumed that the contagious and non-contagious risks are independent from each other. However, considering that the social contagion should eventually influence dietary behaviour and physical activity to achieve a “transmission of obesity” in real life, it should be more natural to account for the dependence between *β* and *ε* (see Discussion). When we numerically solve the system (1), we consider an initial condition with *S*(0) = *N*. Solving equations, d(*S*, *I*, *R*)/d*t* = 0 and analysing the linearized equations, we find an asymptotically stable equilibrium point, (S*, I*, R*) to which all the trajectories of the system converge so that the parameter sensitivity and the age-specific risk in the equilibrium can be examined.

### Lifetime risk of obesity: age-dependence

Although the present study focuses on temporal dynamics of obesity epidemic, here we consider the age-dependent dynamics rather than time-evolution, ignoring time-dependency and measuring only the age-specific risk of obesity in an endemic equilibrium. The age-dependency is specifically considered here, because (i) the most typical epidemiological measurement of obesity at an individual level may be the risk of obesity or associated disease by a certain age (or throughout the course of life), and (ii) we intend to understand the fundamental epidemiological dynamics of obesity using the model (1) as it has direct implication for age-dependent risk of obesity [11].

For simplicity, here we consider an equilibrium state, (S*, I*, R*) with some constant prevalence of obesity. To describe the age-specific risk in a stationary state, we consider variables *X*(*a*) *Y*(*a*) and *Z*(*a*), representing the numbers of never-obese, obese and ex-obese individuals at age *a*, respectively. The dynamics is described as follows:

(2) $$\begin{array}{l} \frac{dX}{da}=-(\lambda^{*}+\mu )X(a),\\ \frac{dY}{da}=\lambda^{*}X(a)+\sigma \lambda^{*}Z(a)-(\mu +\gamma )Y(a),\\ \frac{dZ}{da}=-(\sigma \lambda^{*}+\mu )Z(a)+\gamma Y(a), \end{array}$$

where λ* represents the force of infection which combined both contagious and non-contagious hazards at an equilibrium. The total population size of age *a* is *N*_c_(*a*) = *X*(*a*) + *Y*(*a*) + *Z*(*a*). Due to exponentially distributed life-expectancy of human host, *N*_c_(*a*) is parameterized as

(3) $$N_{c}\left(a\right)=N_{c}\left(0\right)\exp\left(-\mathrm{\mu a}\right),$$

which has been conventionally employed in epidemiology for exploring the age distribution of infected individuals in an endemic equilibrium (Chapter 4 of [11]). Since the total population size remains constant over time, we have

(4) $$N=\int_{0}^{\infty }N_{c}\left(0\right)\exp\left(-\mathrm{\mu a}\right)da=\frac{N_{c}\left(0\right)}{\mu }.$$

In other words, *N*_c_(0) can be equated to *μN*. Since new-borns are assumed as never-obese, we have an initial condition (*X*, *Y*, *Z*) = (*N*_c_(0), 0, 0) and *X*(*a*) is then written as

(5) $$X\left(a\right)=N_{c}\left(0\right)\exp\left\{-\left(\lambda^{*}+\mu \right)a\right\}.$$

We define the life-time risk as a probability of not remaining in the never-obese state throughout the course of life, which is calculated by using the probability to remain never-obese by age *a*, *x*(*a*) = *X*(*a*)/*N*_c_(0). The cumulative risk by age *a*, *q*(*a*), is computed as

(6) $$q\left(a\right)=\int_{0}^{a}\lambda^{*}x\left(s\right)\mathrm{ds}=\frac{\lambda^{*}}{\lambda^{*}+\mu }\left[1-\exp\left\{-\left(\lambda^{*}+\mu \right)a\right\}\right].$$

As *a* → ∞, *q*(*a*) takes λ^*^/(λ^*^ + *μ*). This indicates that, the larger the prevalence, the larger the life-time risk to experience obesity at least once during the course of life. Accordingly, hereafter we use the equilibrium prevalence, calculated from time-dependent system (1), as an epidemiological outcome measure to assess and compare the effectiveness of different interventions.

### Parameter setting

For the exposition of the epidemiological dynamics using time-dependent model, we parameterize model (1) referring to published empirical data. Table 1 summarizes the parameter values. We consider a hypothetical population with a population size *N* = 100,000 which experiences random mixing, with the life expectancy at birth, 1/*μ* = 69.4 years, calculated as the weighted average of country-specific life expectancies [1], which is broadly consistent with those in Southeast Asian countries (e.g. Laos at 62.8 years, Indonesia at 71.6 years and Vietnam at 72.4 years). The relative risk of weight regain among ex-obese individuals, *σ* is set at 8.0 according to literature [12]. The average duration of obesity, 1/*γ* and non-contagious hazard of obesity, *ε* are estimated at 35.8 years and 0.012 per year, respectively, based on the dataset from Framingham Heart Study [13]. The transmission coefficient, *β* is also explicitly estimated from an empirical dataset. Since our model in continuous time is not consistent with empirically observed risk on a static network [4], and because the other data from a social network were sampled from a non-stationary process with non-linear dynamics [13], the dataset for estimating *β* in the present study was derived from a confined household setting. The empirically observed household secondary attack proportion, SAP, has ranged from 0.14 to 0.28 for a short period of time as compared with the life expectancy at birth (e.g. for 4–28 years) [12]. Based on a generalized stochastic epidemic model in the confined setting [14,15], the SAP with a single index case is translated to the basic reproduction number, *R*_0_ by

(7) $$\mathrm{SAP}=\frac{R_{0}}{R_{0}+m},$$

where *m* represents the number of susceptible-and-exposed individuals in the household. In similar epidemic systems, *R*_0_ is mathematically derived from a linearlized system (i.e. nearby disease-free equilibrium) as defined elsewhere [16], but unfortunately, disease-free equilibrium is always unstable for the system (1) except for *ε* = 0 (i.e. except for the case without non-contagious hazard of obesity). Only for now, we use this special case, i.e., *R*_0_ = *βN*/(*γ + μ*), that can only be true and theoretically derived when the non-contagious hazard of obesity is assumed as zero (which is a reasonable assumption for the empirical data based on observation for a short period of time [12]). Assuming that *m* = 3 and SAP ranged from 0.135 to 0.254, the transmission coefficient, *β* in our scenario analysis ranges from 1.99 × 10^-7^ to 4.33 × 10^-7^. The mid-point of estimates, i.e., 2.96 × 10^-7^ is used as the baseline value.

**Table 1:** Parameter values for the transmission model to describe an obesity epidemic

**Description**	**Notation**	**Baseline value**	**Reference**
Population size	*N*	100,000	assumed
Average life expectancy at birth	1/*μ*	69.4 (years)	[1]
Transmission coefficient of obesity	*β*	2.96 × 10^-7^ (per year)	[12]
Non-contagious hazard of obesity	*ε*	0.012 (per year)	[13]
Relative hazard of obesity among ex-obese	*σ*	8.0	[12]
Average duration of obesity	1/*γ*	35.8 (years)	[13]

The parameters shown above were used for baseline scenario. During univariate sensitivity analysis, these parameters were also used except for a single parameter that was varied.

### Computational scenarios

First, we solve the system (1) numerically to understand the time-dependent dynamics of never-obese, obese and ex-obese individuals. Second, we explore the impact of hazard parameters (i.e. hazards for contagious and non-contagious routes) and recovery parameters on the equilibrium prevalence of obesity. Third, to assess and compare the effectiveness of different control programs of obesity, we investigate the sensitivity of the equilibrium prevalence on the shift of parameters that determine the effectiveness of each program. When exploring the effectiveness of interventions, we use two different types of classification of control programs: (i) we consider varying only one parameter for each sensitivity analysis, and (ii) we consider varying a combination of parameters. For the latter, varying a combination of parameters that influence the risk of obesity among never-obese individuals is hereafter referred to as the primary prevention, and varying the other combination of interventions that influence the risk of obesity among obese and ex-obese individuals is referred to as the secondary prevention. It should be noted that the term “secondary prevention” is used here to represent the intervention that happens after experiencing illness (i.e. obesity) at least once. We measure the effectiveness of control programs by examining the impact of relative change in either (i) or (ii) on the equilibrium prevalence value.

## Results

### Baseline dynamics of obesity

**Figure 1:**
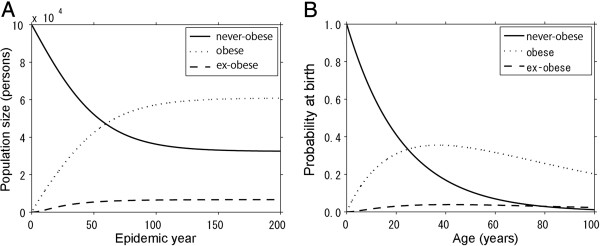
**Baseline dynamics of an obesity epidemic.** (**A**) Time-dependent and (**B**) age-dependent epidemiological trajectories are shown. **A**. The time evolution of the numbers of never-obese, obese and ex-obese individuals. As time goes by, the prevalence of obesity converges to an equilibrium level. **B**. Age-specific risk of obesity in a stationary state. The vertical axis represents the risk (or probability) of age *a* at birth (and thus, it should be noted that the proportions do not sum up to 1 due to natural mortality).

Using aforementioned mathematical model (1), we consider the time evolution of prevalence (Figure 1A). As mentioned above, it should be noted that the initial condition (*S*(0), *I*(0), *R*(0)) = (*N*, 0, 0) is set to demonstrate that obesity-free equilibrium is unstable and the dynamics surely causes an epidemic with an initial fuel from non-contagious hazard. As time goes by, the prevalence converges to a stationary value. According to the baseline setting in Table 1, it takes approximately 200 years to reach to an equilibrium state and the prevalence in our baseline setting is calculated at 60.8%. Although the prevalence estimate is higher than the empirically reported value, the obesity in real world is still growing, and on the technical side, the high value has resulted from exponentially distributed survival. Figure 1B shows the age distribution of *S*, *I* and *R* as a function of age *a*, using system (2) with the equilibrium prevalence and assuming that all new-borns are never-obese. The risk of obesity at a given age *a* (calculated as the “risk at birth”) hits a peak at the age of 37.0 years, but subsequently decreases due to natural mortality.

### Hazard of and recovery from obesity

**Figure 2:**
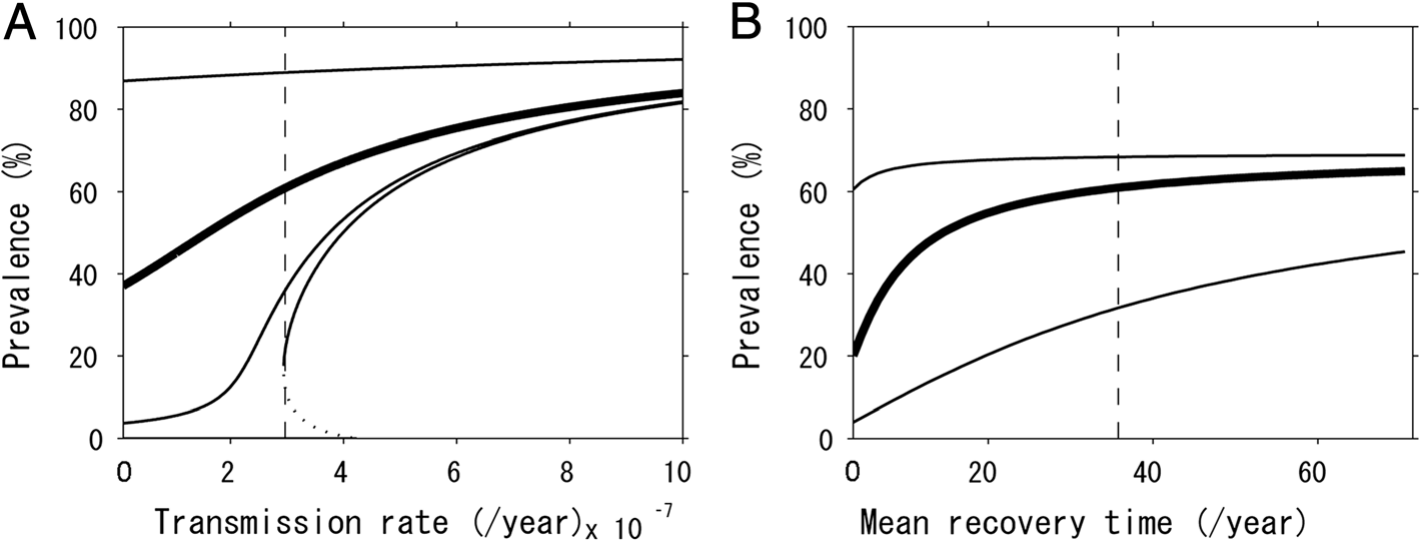
**Sensitivity of the prevalence of obesity to the parameters determining the hazard and the recovery.** Equilibrium prevalence is computed by varying a single parameter, i.e., the transmission rate for panel A and the mean recovery rate for panel B. **A**. The bold line shows the baseline result, varying only the transmission coefficient *β*. The other lines represent the scenarios in which *ε* is varied to the 0%, 10% and 1000% relative to the baseline value (from the horizontal axis to the top, the lines represent 0, 10 and 1000%, respectively). **B**. The bold line shows the equilibrium prevalence of obesity using baseline parameter values other than the average duration of obesity, 1/*γ.* Two other lines represent the scenarios in which *σ* is varied to 10% (bottom) and 1000% (top), respectively, relative to the baseline value.

Obviously, the contents, subjects and objectives of many available interventions differ by control programs. Theoretically speaking, there are two types of interventions that belong to the primary prevention, i.e. the intervention on social contact and the preventing weight gain among never-obese individuals, each influencing the transmission coefficient *β* and non-contagious hazard *ε*, respectively. The intervention of social contact is intended to prevent person-to-person transmission by suppressing obesity contagion. The practical feasibility of such an intervention is subject to discussion, but in the present study the intervention is theoretically considered as resembling contact tracing of directly transmitted infectious diseases [17,18]. Preventing weight gain among never-obese individuals is to control the diet and enhance physical activities, including the specification of nutrients and restriction of calorie intake [19]. Figure 2A shows the role of *β* and *ε* in regulating the prevalence of obesity. Overall, the lower the transmission coefficient *β* is, the lower the prevalence would be. However, the equilibrium prevalence appears to be very sensitive to *β*, and abruptly varies at some value of *β* depending on the non-contagious hazard *ε*. For instance, when *ε* was set as equal to 0, one could theoretically expect an eventual eradication of obesity by controlling obesity contagion, and in such an instance, a disease-free equilibrium could occur. In this case, the model also appears to yield a backward bifurcation of prevalence, indicating the absence of simple threshold governed by *β*. That is, due to the presence of re-infection, the model can find an endemic equilibrium even for *R*_0_ < 1, indicating a difficulty in controlling obesity in the presence of person-to-person transmission.

There are two types of interventions that belong to the secondary prevention, i.e. the dietary control program among obese individuals and the follow-up program of ex-obese individuals, each influencing on the duration of obesity 1/*γ* and the relative hazard among ex-obese *σ*, respectively. The dietary restriction in this context is targeted on obese individuals only [20], and the follow-up program is to encourage ex-obese individuals not to be overweight again [21]; ex-obese individuals are known to be more prone to obesity than never-obese individuals [22]. Figure 2B shows the role of 1/*γ* and *σ* in regulating the prevalence of obesity. Overall, the shorter the duration of obesity 1/*γ* is, the lower the equilibrium prevalence would be. Unlike Figure 2A, the prevalence does not abruptly vary with *σ*. Varying *σ* to lower or greater values led the prevalence of obesity to be less sensitive to 1/*γ*.

### Comparison of intervention effectiveness

When we compare the effectiveness of multiple control programs, interventions that vary only a single parameter of model (1) are separately examined from those varying a combination of multiple parameters. For the combination of multiple parameters, the primary and secondary preventions are separately grouped for comparison due to practical consistency in the grouping. Since the system (1) focused on the intrinsic dynamics without any interventions, here we specifically show the way that extrinsic factors influence the growth of obesity. As a parameter governing the primary prevention *α*, we assume that both contagious and non-contagious hazards are equally reduced by the factor *α* as follows:

(8) $$\begin{array}{l} \frac{dS}{dt}=\mu N-\alpha [\beta I(t)+\varepsilon ]S(t)-\mu S(t),\\ \frac{dI}{dt}=\alpha [\beta I(t)+\varepsilon ]S(t)+\sigma [\beta I(t)+\varepsilon ]R(t)-\left(\mu +\gamma \right)I(t),\\ \frac{dR}{dt}=\gamma I(t)-\sigma [\beta I(t)+\varepsilon ]R(t)-\mu R(t). \end{array}$$

It should be noted that only the hazards among never-obese individuals are reduced. In addition, there is a possibility that an assumed marginal independence between *β* and *ε* could lead to an overestimation of the effectiveness of primary prevention (because the reduction of prevalence with an identical *α* in the presence of dependence can be greater than that we show here). Similarly, we consider the secondary prevention which includes the dietary restriction among obese individuals and the follow-up program among those experienced obesity at least once in combination. Supposing that the associated intervention programs are enhanced by a factor *κ*, we modelled the secondary prevention as follows:

(9) $$\begin{array}{l} \frac{dS}{dt}=\mu N-[\beta I(t)+\varepsilon ]S(t)-\mu S(t),\\ \frac{dI}{dt}=[\beta I(t)+\varepsilon ]S(t)+\kappa \sigma [\beta I(t)+\varepsilon ]R(t)-\left(\mu +\kappa \gamma \right)I(t),\\ \frac{dR}{dt}=\kappa \gamma I(t)-\kappa \sigma [\beta I(t)+\varepsilon ]R(t)-\mu R(t). \end{array}$$

It should be noted that the follow-up program reduced the overall hazard of re-infection (including those arising from social contagion and lifestyle), because the follow-up program does not specify the way of regaining weight among ex-obese individuals and is primarily intended to reduce susceptibility of ex-obese individuals toward re-infection.

**Figure 3:**
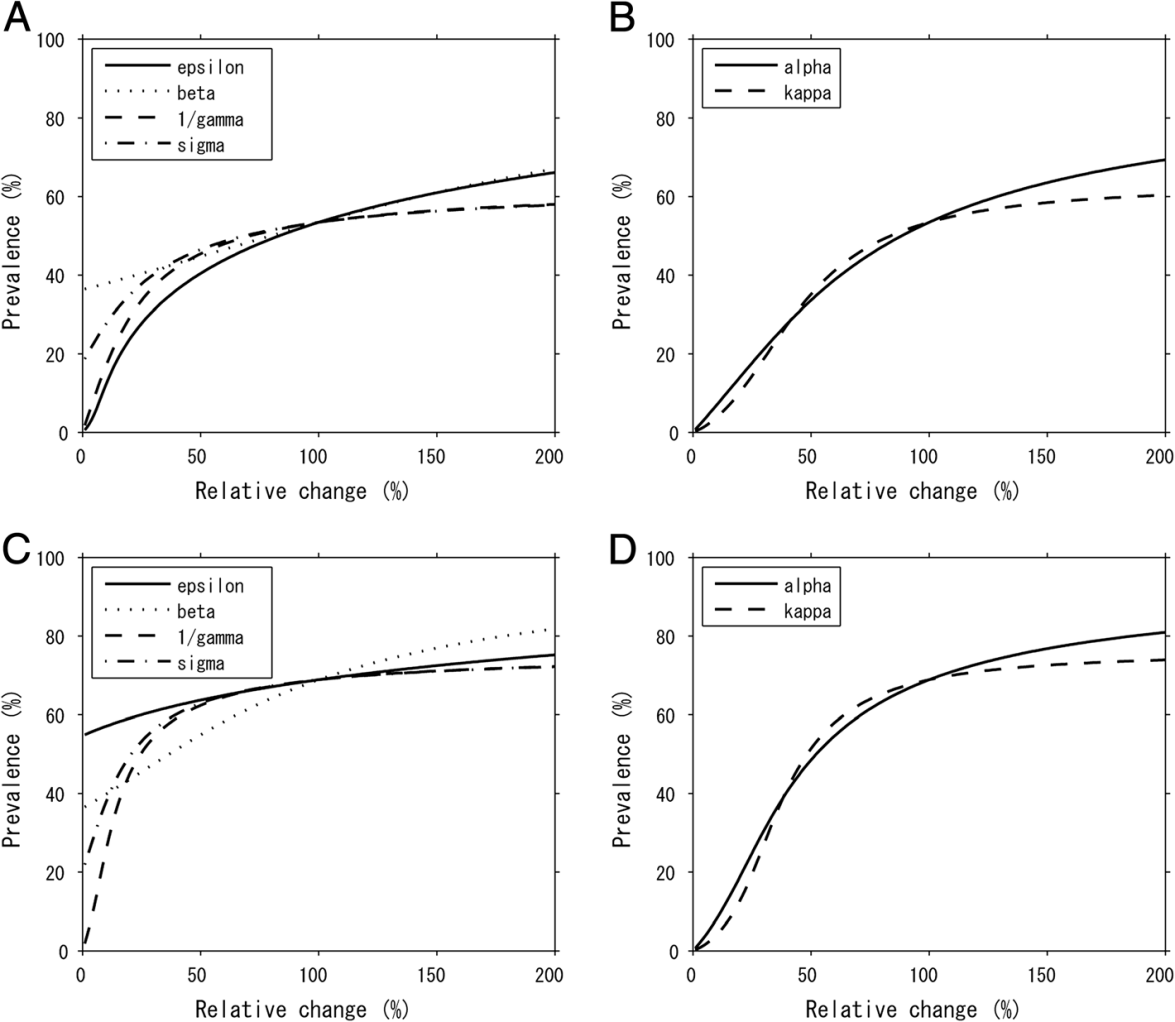
**Sensitivity of the prevalence of obesity to different control programs.** Effectiveness of interventions is measured by equilibrium prevalence as a function of relative reduction in certain parameter(s). **A **&**C**. The prevalence when single parameters (*ε*, *β*, 1/*γ* and *σ*) are independently varied. **B** &**D**. Comparison between the primary prevention (reducing *α*) and the secondary prevention (reducing *κ*). The baseline value of *β* is set to be low in panels **A** and **B** (1.99 × 10^-7^ per year), while panels **C** and **D** shows the case when *β* is set at high (4.33 × 10^-7^ per year).

Figure 3 shows the sensitivity of prevalence to independent variations in each parameter. While panels A and C show the results of univariate sensitivity, panels B and D are the results from varying two parameters in combination. Panels A and B employ 1.99 × 10^-7^ per year as the transmission coefficient *β* which is the lowest in range, while Panels C and D adopted the highest value 4.33 × 10^-7^ per year. When *β* is small, Figure 3A demonstrates that preventing weight gain among never-obese individuals, *ε*, is most effective and influential. Dietary restriction among obese individuals, 1/*γ*, appeared to be the second most effective option. Namely, as long as *β* remains very small, and thus, the transmission of obesity cannot be maintained in the host population via person-to-person transmission routes, an intervention program that aims to reduce the non-contagious hazard would be the most effective strategy, and moreover, quickly removing obese individuals by the control program would be expected to reduce obesity effectively. Combined interventions are compared in Figure 3B. The effectiveness of reducing overall hazards of obesity among never-obese individuals would be similar to that of targeting obese and ex-obese individuals. In addition, increasing *α* would be more influential to elevate the prevalence than increasing *κ*.

However, when the transmission coefficient is set to be very high so that the transmission of obesity can be maintained through social contagion, preventing weight gain among never-obese individuals, *ε*, appears to be the least effective. Rather, promoting the dietary restriction (1/*γ*) and implementing the follow-up program (*σ*) would be more effective in reducing the prevalence of obesity. In a certain range, intervening *β* is the most influential parameter in reducing the prevalence, while in reality it might be difficult to directly reduce obesity contagion by a control program. When a combination of two control strategies can be selected, the primary and secondary preventions yielded similar population impacts and the superiority of the effectiveness depends on the strength of the interventions at an individual level.

## Discussion

In the present study, we investigated epidemiological models that describe the obesity epidemic, spreading via social contact and acquired due to non-contagious reasons. We assessed and compared the effectiveness of different types of intervention programs which aim to reduce the risk of obesity. As the most important practical finding, we identified that the optimal choice of intervention programs considerably varies with the transmission coefficient of obesity, *β*. When *β* is small, the transmission cannot be maintained by social contagion alone. In such an instance, our model has suggested that preventing weight gain among never-obese individuals would be the most effective option, although it should be remembered that our approach adopted marginal independence between *β* and *ε*, and the effectiveness of primary prevention might have been overestimated. When *β* is large enough to sustain the transmission of obesity through the person-to-person route, dietary restriction among obese individuals could potentially be the most effective. In other words, depending on the transmissibility of obesity, the effectiveness of reducing obesity hazards would greatly vary, and thus, the population impact of each program would be dependent on the transmission dynamics of obesity. When a combination of interventions can be selected, the primary prevention is likely more influential than the secondary prevention for a small effect size, but on the whole primary and secondary preventions yielded similar population impacts. Despite the dependence of optimal interventions on *β*, it should be noted that the transmission potential of obesity in community setting has yet to be explicitly estimated.

Since WHO has addressed DPAS, emphasizing the importance of diet and physical activity as two main factors that determine the risk of obesity [3], the worldwide effort of obesity control has started, conducting and evaluating various programs. As we have shown using a simplistic model, the social contagion of obesity must be a key concern for public health for decision-making, because the design of effective control programs requires us to capture and understand the population dynamics of obesity in an explicit manner, and moreover, empirically quantify the transmissibility of obesity. As the most important data gap, we have identified that the transmission potential of obesity contagion has to be estimated, as it drastically varies the optimal choice of interventions. To estimate the contagious hazard of obesity, household-based prospective cohort study of susceptible and recovered individuals is desirable, because not only the transmissibility within households but also the relevance of the transmission potential to the natural history of obesity can be measurable. Nevertheless, it should be noted that the threshold property using *R*_0_ is unlikely to be useful in the obesity model due to non-contagious risk and re-infection.

Whereas we have shown that primary and secondary preventions yielded similar reductions in the equilibrium prevalence of obesity in a certain parameter space, it should be remembered that the primary and secondary preventions require different types and amounts of effort, not sharing an identical effect size. Considering that the length of obese period could influence the risk of later health outcomes (e.g. diabetes), the primary prevention may better be more advantageous in reducing the devastating outcomes. Addressing the associated life-course issues including an assessment of economic impact is the subject for future studies.

Despite our key finding in identifying the transmissibility as the most influential component to determine the optimal interventions, there are five issues that are regarded as limitation or should be cautiously interpreted. First, while obesity contagion on a social contact network has been empirically studied in literature [22-24], we have employed a homogeneously mixing assumption for mathematical convenience and to identify key parameter of obesity dynamics without ambiguity [25]. Of course, using empirically observed network data would permit us to describe more realistic situations [13]. Considering that the threshold level of obesity epidemic likely differs in heterogeneous contact networks, future studies should quantify the transmissibility of obesity on an explicit contact network and identify the corresponding appropriate way of public health control. Second, the natural history of obesity, including the duration of obesity and frequency of recurrence, is largely unknown [26]. Due to shortage of information, we have had to ignore age-dependent heterogeneity, e.g. differential calorie consumptions by age [27]. Third, an equilibrium prevalence of our model is calculated as high as 60.8%, which is greater than currently observed prevalence [1]. However, the prevalence in the present day has yet to reach the stable level, and has been in increasing trend [13], and thus, we believe that our exercise has not been far from reality even by using a simplistic model. Fourth, we did not take into account the cost to be compared across different intervention programs. Identification of optimal programs would require an explicit analysis of cost-benefit and cost-effectiveness aspects. Lastly, on the technical side, further work could explore the use of alternative modelling approaches, e.g. conditional risk model with stochastic dependence structure between risks with different routes of obesity, which could avoid overestimating the effectiveness of primary prevention.

Despite these limitations due mainly to simplifications of our modelling exercise, a number of advantages in our study should be noted. First, we took into account the relative hazard of obesity among ex-obese individuals, while an earlier study that shares a similar scopes with our study ignored the elevated risk of weight regain among ex-obese individuals [13]. Rather than focusing on network heterogeneity, our study has intended to examine the impact of nonlinear transmission with complex natural history on the optimal choice of interventions [28,29]. Second, due to simple model structure, our model has remained to be analytically tractable, and thus, a variety of different epidemiological measures, including life-time risk of obesity-related diseases, can be additionally derived. For instance, one can easily extend our concept to account for the delay or a fraction of obese individuals in developing a chronic disease later in life. Using a convolution of the time delay function from obesity to a heart attack, *f*(*s*) of length *s* and the risk of once becoming obese by age *a*, 1-*x*(*a*), with a scaling factor (i.e. the overall risk) of heart attack *p*, one can describe the risk of heart attack as a function of age as

(10) $$w\left(a\right)=p\int_{0}^{\infty }f\left(s\right)\left(1-x\left(a-s\right)\right)ds,$$

although the use of 1-*x*(*a*) is subject to discussion (e.g. rather, one may prefer to use individual history of being obese). Such modelling exercise can potentially enable us to describe the long-term and secondary impact of obesity control in reducing closely associated diseases or deaths at a population level, while explicitly accounting for nonlinearity in the spread and control of obesity. Third, a little more complex natural history may better be incorporated into the model. For instance, non-contagious hazard was assumed as a fixed value in the present study, but in reality the hazard may depend on age which reflects not only the physiological age-dependence but also the history of escape from obesity. Not only obesity but also other behavioural contagion can be analysed using similar modelling approaches [30]. Despite numerous future tasks, we believe that we have successfully simplified the population dynamics of obesity, identifying the importance of quantifying the transmission potential to determine public health control programs in the future.

## Conclusions

The optimal choice of interventions against obesity varies by the transmission potential of obesity from person to person. To attain appropriate assessment and comparison of different types of public health control programs of obesity, it is critical that the epidemiological dynamics of obesity, especially the transmission potential, is quantified in advance.

## Competing interests

The authors declare that they have no competing interests.

## Authors’ contributions

HN conceived the study idea and KE and HN constructed the epidemiological model. KE implemented numerical analysis with supervision from HN. KE and HN jointly drafted the manuscript. KA gave comments and advice on the earlier version of the manuscript. All authors approved the final version of the manuscript.
